# De novo variants in immune regulatory genes in Down syndrome regression disorder

**DOI:** 10.1007/s00415-024-12521-y

**Published:** 2024-06-22

**Authors:** Saba Jafarpour, Abhik K. Banerjee, Mellad M. Khoshnood, Benjamin N. Vogel, Natalie K. Boyd, Lina Nguyen, Rebecca Partridge, Stephanie L. Santoro, Grace Y. Gombolay, Kristen S. Fisher, Diego Real de Asua, Maria Carmen del Ortega, Cathy Franklin, Michael S. Rafii, Jonathan D. Santoro

**Affiliations:** 1 Division of Neurology, Department of Pediatrics, Children’s Hospital Los Angeles, 4650 Sunset Blvd, Mailstop 82, Los Angeles, CA 90027 USA; 2 Department of Neurology, Keck School of Medicine of the University of Southern California, Los Angeles, CA USA; 3 Los Angeles General Hospital, Los Angeles, CA USA; 4 Virginia Mason Health System, Issaquah, WA USA; 5 Department of Genetics, Harvard Medical School, Boston, MA USA; 6 Down Syndrome Program, Massachusetts General Hospital, Boston, MA USA; 7 Division of Neurology, Department of Pediatrics, Emory School of Medicine, and Children’s Healthcare of Atlanta, Atlanta, GA USA; 8 Department of Neurology, Baylor College of Medicine, Houston, TX USA; 9 Adult Down Syndrome Outpatient Clinic, Department of Internal Medicine, Fundación de Investigación Biomédica, Hospital Universitario de La Princesa, Madrid, Spain; 10 Department of Psychiatry, Clinica Universidad de Navarra, Madrid, Spain; 11 Department of Psychiatry, Mater Research Institute, University of Queensland, Brisbane, Australia

**Keywords:** Down syndrome, Regression, Interferon, Variant, Gene

## Abstract

**Background:**

Down Syndrome Regression Disorder (DSRD) is a rare and poorly understood disorder of the central nervous system, characterized by acute or subacute neuropsychiatric symptoms in previously healthy individuals with Down syndrome (DS). Many patients exhibit immunotherapy-responsiveness, indicative of immune dysregulation as a potential underlying etiology. While hypotheses are emerging regarding the role of interferon signaling in DSRD and other autoimmune conditions associated with DS, it is unclear why a small subset of individuals with DS develop DSRD. The aim of this study was to investigate genes of immune regulation in persons with DSRD.

**Methods:**

This study included individuals with DSRD aged 10–30 years with trio exome sequencing performed during the diagnostic work up. Descriptive statistics and univariate analysis (Chi-square and Fisher’s exact test) were used to describe and compare the characteristics of individuals with and without variants.

**Results:**

Forty-one individuals with DSRD had trio exome sequencing results. Eight (20%) had heterozygous de novo variants of immune regulatory genes, with four variants being pathogenic or likely pathogenic (*UNC13D, XIAP, RNASEH2A, and DNASE1L3*). All genes harboring pathogenic variants were associated with interferon type-1 inflammatory response. Individuals harboring variants were more likely to have a preceding trigger (*p* = 0.03, 95% CI 1.21–97.06), rapid clinical decline in less than 1 month (*p* = 0.01, 95% CI 1.67–52.06), and MRI abnormalities (*p* < 0.001, 95% CI 4.89–527.71).

**Discussion:**

A distinct subset of individuals with DSRD exhibited pathogenic variants in immune regulation genes associated with interferon-mediated inflammatory response, coinciding with previously established links between these genes and interferonopathies such as Aicardi-Goutieres syndrome. Our observations suggest that these variants might potentially contribute to the development of DSRD in individuals with DS.

**Supplementary Information:**

The online version contains supplementary material available at 10.1007/s00415-024-12521-y.

## Introduction

Down syndrome (DS) is one of the most common genetic disorders, with an incidence of 1 in every 800 live births in the United States [1]. An emerging condition, Down Syndrome Regression Disorder (DSRD), has been reported with increasing frequency over the last 2 decades [2–4]. DSRD is an acute or subacute neuropsychiatric disorder which primarily occurs in individuals with DS between ages 10 and 30 years [5]. It includes symptoms of bradykinesia, catatonia, developmental regression, encephalopathy, insomnia, mutism, and loss of ability to perform activities of daily living [2–4, 6–8]. This condition is often severe and can significantly impact the quality of life and autonomy of persons with DS.

The etiology of DSRD is not clear. According to several multi-center studies, as high as 85% of individuals with DSRD have been reported to respond to immunotherapy [5–7, 9]. There is a higher likelihood of immunotherapy responsiveness in individuals with neurodiagnostic abnormalities (e.g., abnormal MRI or cerebrospinal fluid studies) though the precise origin of these abnormalities and their link to immunotherapy response remain unclear [6]. Individuals with DS are reported to have other co-occurring genetic and autoimmune conditions [10]. Several studies have examined downstream immune dysregulation in individuals with DS and have hypothesized that amplified interferon signaling secondary to increased interferon receptor gene dosage (due to trisomy of chromosome 21) as a major contributor [11–13]. While hypotheses are emerging regarding the role of interferon signaling in DSRD and other autoimmune conditions associated with DS, it is unclear why a small subset of individuals with DS develop DSRD.

Susceptibility to polygenic diseases stem partially from variations in environmental stressors and socioeconomic factors. However, genetic variations also play a significant role. Traditionally, the quest to identify genetic variants linked to complex or polygenic traits and diseases has relied on genome-wide association studies (GWAS), which identify common variants (i.e., single nucleotide polymorphisms, SNP). However, rare variants with potentially larger effect-size are not identified through GWAS. In addition, variants identified through GWAS often localize to non-coding regions, complicating results interpretation [14]. Identification of rare variants in the coding regions could provide insights into the underlying pathophysiological mechanisms, particularly for conditions where a clear etiology has yet to be established.

This study sought to evaluate contribution of rare variants within coding regions of genes related to immune regulation to DSRD. Additionally, we aimed to investigate whether these variants are associated with specific clinical characteristics such as disease severity, abnormality in neurodiagnostic studies, or responsiveness to immunotherapy.

## Methods

### Patient population and approvals

A retrospective, chart-based, review was performed of individuals enrolled in the DS Neurology program at the Children’s Hospital Los Angeles (CHLA) between July 1st, 2019, and October 1st, 2023. This included patients physically evaluated at the primary center or those with whom chart review was performed in secondary consultation with an outside physician. Institutional Review Board (IRB) approval was obtained through CHLA and the University of Southern California, and consent and assent were waived for this study as the clinical data reviewed had already been obtained.

### Inclusion criteria

All individuals in both cohorts required a genetically confirmed diagnosis of Trisomy 21. Individuals with either possible or probable DSRD per international consensus criteria were included [15]. Further, all individuals had to be aged 10–30 years at the time of symptom onset to qualify for the study. Individuals in the genetic testing cohort required trio testing (biological parents) to assess if variants were de novo.

### Exclusion criteria

Individuals who had translocation or mosaic forms of DS were excluded. Further, if an individual was found to have an alternative explanation for their symptoms (e.g., cerebrovascular accident or epilepsy), that participant was excluded.

### Data collection

Data were collected through retrospective review of the electronic medical record (EMR). Demographic data, medical and surgical history, along with clinical findings and diagnostic results, were obtained from clinical records. Any potential triggers linked to the onset of symptoms were noted, including infection, change of school or home environment, loss of care giver or friend, death in family, physical or emotional abuse, or any medical events. These factors were considered potentially contributory if they occurred within 12 weeks before the onset of symptoms. All individuals included in this study had received Bush Francis Catatonia Rating Scale (BFCRS) [16] and Neuropsychiatric Inventory-Questionnaire (NPI-Q) [17] at baseline and 24 weeks following immunotherapy initiation. Immunotherapy consisted of intravenous immunoglobulin (IVIg) and/or steroid administration. Immunotherapy-responsiveness was defined as a 50% or more improvement on BFCRS or NPIQ scores.

### Neurodiagnostic abnormalities

EEG abnormality was defined as focal or generalized slowing, focal or generalized epileptiform discharges out of any cortex, or seizure. MRI had to be performed with and without contrast administration on a 3 T scanner. All abnormal findings except for structural malformations (e.g., Chiari malformation) were categorized as abnormal. Any of the following cerebrospinal fluid (CSF) findings were considered abnormal: WBC count > 5 cells/mm^3^, total protein > 60 mg/dL, presence of oligoclonal bands, an IgG index of > 0.66, and/or an elevated neopterin (> 33 nmol/mL). The presence of over 1000 RBC in the CSF indicated hemorrhagic contamination and, therefore, the results were excluded from analysis [6].

### Next generation sequencing and bioinformatic analysis

Next-generation sequencing of exomes was performed. Variants of uncertain significance were first analyzed using Varsome Human Genomic Variant Search Engine and the hg38 genome assembly [18]. The Single Nucleotide Polymorphism Database (dbSNP) reference SNP ID number (rs number) was reported when available. Variant frequency was adapted from Varsome gnomAD Exome v2.1.1. Conservation scores were adapted from PhyloP100 scores available on Varsome browser. Genomic location was taken from the Varsome Variant Tab and used to calculate Combined Annotation-Dependent Depletion (CADD) scores using the Single Nucleotide Variant Lookup tool and the GRCh38-v1.6 CADD model [19]. Maximum CADD scores were reported.

For in silico prediction of variant function, we first downloaded FASTA sequences for each protein using the National Library of Medicine’s protein search. FASTA sequences were then analyzed using Polymorphism Phenotyping v2 (PolyPhen-2) and Sorting Intolerant from Tolerant (SIFT) Sequence tool [20, 21]. For meta in silico prediction, we used the meta scores available on Varsome browser, based on the aggregated evidence from different in silico predictors (MetaRNN, REVEL, BayesDel noAF, BayesDel addAF, MetaLR, MetaSVM) [18].

### Variant calling

Interpretation of variants were performed according to the joint consensus recommendation of the American College of Medical Genetics (ACMG) and Genomics and the Association for Molecular Pathology (AMP) [22]. Results were interpreted by a board-certified clinical molecular geneticist. For quantitative variant classification, a naturally converted Bayesian formulation point system was used [23].

### Pathway analysis

To perform Kyoto Encyclopedia of Genes and Genomes (KEGG) pathway analysis using clinical diagnosis gene lists, lists of gene names were first imported into the NIAID/NIH Database for Annotation, Visualization and Integrated Discovery (DAVID) Bioinformatics Resources v.6.8 Analysis Wizard Tool. “OFFICIAL_GENE_SYMBOL” was selected in the Identifier field, Homo sapiens was inputted within the Species field, and “Gene List” was selected under List Type. Next, the imported gene list was analyzed using the DAVID Functional Annotation Tool set, specifically looking within the “Pathway” and “KEGG_Pathway” tools [24, 25].

### Statistical analysis

Descriptive statistics were used to summarize the characteristics of patients included in this study. Univariate analyses (Chi square and Fisher’s exact test) were used to compare the characteristics of individuals with and without variants. For KEGG pathway analysis, *p* values and Benjamini corrections were calculated. Benjamini values of < 0.05 were considered statistically significant. Analyses were performed using DAVID Bioinformatics Resources 6.8.

## Results

Of the 347 individuals with DSRD identified for inclusion, 41 (12%) had trio exome sequencing performed during their diagnostic work up. Demographic and clinical features of the cohort are reported in Table 1. A high percentage of individuals with exome sequencing testing had commercial insurance (35/41, 85%) which was significantly higher than the rate of commercial insurance in our comparator DSRD cohort (159/306, 52%) (*p* ≤ 0.001, 95% CI 2.20–13.19). There were no other statistically significant differences with regards to the DSRD cohort without exome sequencing.

**Table 1:** Demographics and clinical characteristics of individuals with DSRD with and without exome sequencing results

	DSRD with exome sequencing (*n* = 41)	DSRD without exome sequencing (*n* = 306)	*p* value	95% CI
Demographics				
Sex				
Male	22 (54%)	160 (52%)	0.87	0.55–2.03
Female	19 (46%)	146 (48%)		
Race				
White	31 (76%)	244 (80%)		
Black/African-American	4 (10%)	27 (9%)	0.54	0.37–1.69
Asian	4 (10%)	22 (7%)		
Other	2 (4%)	13 (4%)		
Ethnicity				
Hispanic or Latino	17 (41%)	168 (55%)	0.11	0.30–1.13
Not Hispanic or Latino	24 (59%)	138 (45%)		
Commercial insurance	35 (85%)	159 (52%)	< 0.001*	**2.20–13.19**
Age at symptom onset (median, IQR)	14 (12–16)	14 (12–16)	0.59	0.87–1.37
Age at diagnosis (median, IQR)	17 (15–21)	18 (16–22)	0.42	0.49–3.62
Congenital heart disease	16 (39%)	134 (44%)	0.56	0.42–1.60
Non-DSRD autoimmune disease	20 (49%)	125 (41%)	0.34	0.72–2.65
Prior diagnosis of ASD	1 (2%)	7 (2%)	0.95	0.13–8.91
Clinical features				
Trigger present	22 (54%)	149 (49%)	0.55	0.63–2.35
Infection	10 (45%)	63 (42%)		
Change of school/home environment	2 (9%)	13 (9%)		
Loss of caregiver/friend	3 (14%)	15 (10%)		
Death in family	1 (5%)	9 (6%)		
Physical or emotional abuse	4 (18%)	33 (22%)		
Medical event	2 (5%)	16 (11%)		
Months to symptom peak (median, IQR)	3 (1–4)	3 (2–5)	0.89	0.55–1.37
Serum cytokine abnormalities	11 (27%)	70 (23%)	0.57	0.59–2.59
EEG abnormal	12 (29%)	73 (24%)	0.45	0.64–2.72
MRI abnormal	11 (27%)	95 (31%)	0.58	0.39–1.69
Lumbar puncture abnormal	7 (17%)	61 (20%)	0.67	0.35–1.96
Catatonia	31 (76%)	235 (77%)	0.87	0.44–2.01
Immunotherapy responsive	33 (80%)	222 (73%)	0.28	0.69–3.52
BFCRS score > 20 at baseline	14 (34%)	79 (26%)	0.26	0.74–2.98

*ASD* Autism spectrum disorder, *BFCRS* Bush-Francis catatonia rating scale, *CI* confidence interval, *DSRD* Down Syndrome Regression Disorder, *EEG* electroencephalogram, *IQR* inter-quartile range, *MRI* magnetic resonance imaging

*Bolded text is statistically significant value

Of all individuals with exome sequencing testing, eight (20%) were identified as having de novo heterozygous variants classified as pathogenic, likely pathogenic or uncertain clinical significance. Variants of eight immune regulatory genes were detected (Table 2). Of the eight variants identified, four variants were classified as pathogenic (*RNASEH2A:* NM_006397.3:c.557G>A) or likely pathogenic (*UNC13D:* NM_199242.3:c.652G>T,* XIAP:* NM_001378592.1:c.655G>A*,* and *DNASE1L3:* NM_004944.4:c.581G>A)*.* Tables S1 and S2 summarize clinical features and neurodiagnostic findings in individuals with variants. On KEGG pathway analysis, the identified enriched pathway (NOD2 receptor signaling) did not reach statistical significance.

**Table 2:** Variants identified in study population (*n* = 8)

Gene	Variant	dbSNP	gnomAD frequency	PolyPhen	SIFT	PhyloP conservation	CADD	Meta in silico	Classification point [23]	Variant classification
*BAZ1A *(HGNC: 960)	NM_013448.3: c.3278G>A (p.Arg1093Gln)	rs776556963	0.00000406	Probably damaging (score 1.000)	Affect protein function^a^	7.602	28.7	N/A	5	Uncertain
DNASE1L3 (HGNC:2959)	NM_004944.4: c.581G>A (p.Cys194Tyr)	N/A	N/A	Probably damaging (score 1.000)	Tolerated	7.905	26.7	PP3-pathogenic	8	Likely pathogenic
*IRF7 *(HGNC: 6122)	NM_001572.5: c.1405T>C (p.Trp469Arg)	rs746725871	0.0000203	Benign	Tolerated	0.689	22.8	BP4-benign	2	Uncertain
*LYST *(HGNC: 1968)	NM_000081.4: c.1676G>A (p.Arg559His)	rs138011756	0.0000997	Benign	Affect protein function^a^	2.957	23	BP4-benign	0	Uncertain
RNASEH2A (HGNC: 18,518)	NM_006397.3: c.557G>A (p.Arg186Gln)	rs753679297	0.00000398	Probably damaging (score 1.000)	Affect protein function	8.779	29.5	PP3-pathogenic	19	Pathogenic
*SMARCAL1 *(HGNC:11,102)	NM_014140.4: c.488C > A (p.Thr163Asn)	rs748188404	0.0000358	Benign	Tolerated	-1.076	1.35	BP4-benign	1	Uncertain
UNC13D (HGNC: 23,147)	NM_199242.3: c.652G>T (p.Gly218Trp)	rs775666284	0.00000398	Possibly damaging (score 0.932)	Affect protein function^a^	4.593	26.5	PP3-pathogenic	7	Likely pathogenic
XIAP (HGNC: 592)	NM_001378592.1: c.655G>A (p.Glu219Lys)	N/A	N/A	Probably damaging (score 1.000)	Affect protein function	9.339	36	PP3-pathogenic	8	Likely pathogenic

^a^Low confidence call

Across all gene variants identified, no individual harboring a variant exhibited a clinical phenotype consistent with a non-DSRD clinical disorder associated with the gene of concern (e.g., Aicardi-Goutières syndrome). However, individuals with identified variants were noted to have differences in clinical characteristics compared to individuals with no variants (Table 3). Notably, individuals with variants were more likely to have a preceding trigger (*p* = 0.03, 95% CI 1.21–97.06), rapid clinical decline with nadir in less than 1 month (*p* = 0.01, 95% CI 1.67–52.06), and MRI abnormalities (*p* < 0.001, 95% CI 4.89–527.71). The type of trigger (e.g., temporally associated environmental, social, or infectious event) was not associated with a higher likelihood of harboring a variant (*p* = 0.63, 95% CI 0.37–1.96). Other clinical factors such as abnormality of EEG, CSF or serum cytokine panel were not statistically significant nor was the presence of catatonia, BFCRS > 20 or immunotherapy-responsiveness.

**Table 3:** Clinical features of DSRD in individuals harboring a variant in an immune regulatory gene compared to individuals with DSRD without variants

	With variants (*n* = 8)	Without variants (*n* = 33)	*p* value	Odds ratio	95% CI
Trigger present	8 (100%)	16 (48.5%)	0.03*	**10.9**	**1.21–97.06**
Peak symptoms < 1 month from onset	5 (63%)	5 (15%)	**0.01**	**9.3**	**1.67–52.06**
Serum cytokine abnormalities	4 (50%)	13 (39.3%)	0.06	4.8	0.94–24.95
EEG abnormal	2 (25%)	8 (24.2%)	0.97	0.9	0.17–5.59
MRI abnormal	7 (88%)	10 (30.3%)	** < 0.001**	**50.8**	**4.89–527.71**
CSF abnormal	3 (34%)	6 (18.1%)	0.1	4.35	0.74–25.60
Catatonia	6 (75%)	25 (75.7%)	0.96	0.96	0.16–5.74
Immunotherapy responsive	8 (100%)	24 (72.7%)	0.41	2.56	0.28–23.72
BFCRS > 20 at baseline	3 (34%)	14 (42%)	0.80	0.81	0.17–3.99

*BFCRS* Bush-Francis Catatonia Rating Scale, *CI* confidence interval, *CSF* cerebrospinal fluid, *DSRD* Down syndrome regression disorder, *EEG* electroencephalogram, *MRI* magnetic resonance imaging

*Bolded text indicates statistically significant value

## Discussion

This study represents the first extensive examination of exome sequencing in individuals with DSRD. The authors identified heterozygous de novo variants in immune regulation genes in 20% of individuals with DSRD, including pathogenic or likely pathogenic variants in *DNASE1L3*, *RNASEH2A, UNC13D,* and *XIAP* in 10% of cases. Among those with variants, a notable pattern emerged, indicating a higher likelihood of preceding triggers, a more rapid clinical decline, and higher rate of neuroimaging abnormalities. Individuals with these gene variants were previously healthy and did not exhibit the previously recognized conditions associated with pathogenic variants in *RNASEH2A, UNC13D, XIAP, or DNASE1L3*. There was no significant difference in immune therapy responsiveness between individuals with and without variants, although all individuals with variants were immune therapy responsive.

**Fig. 1:**
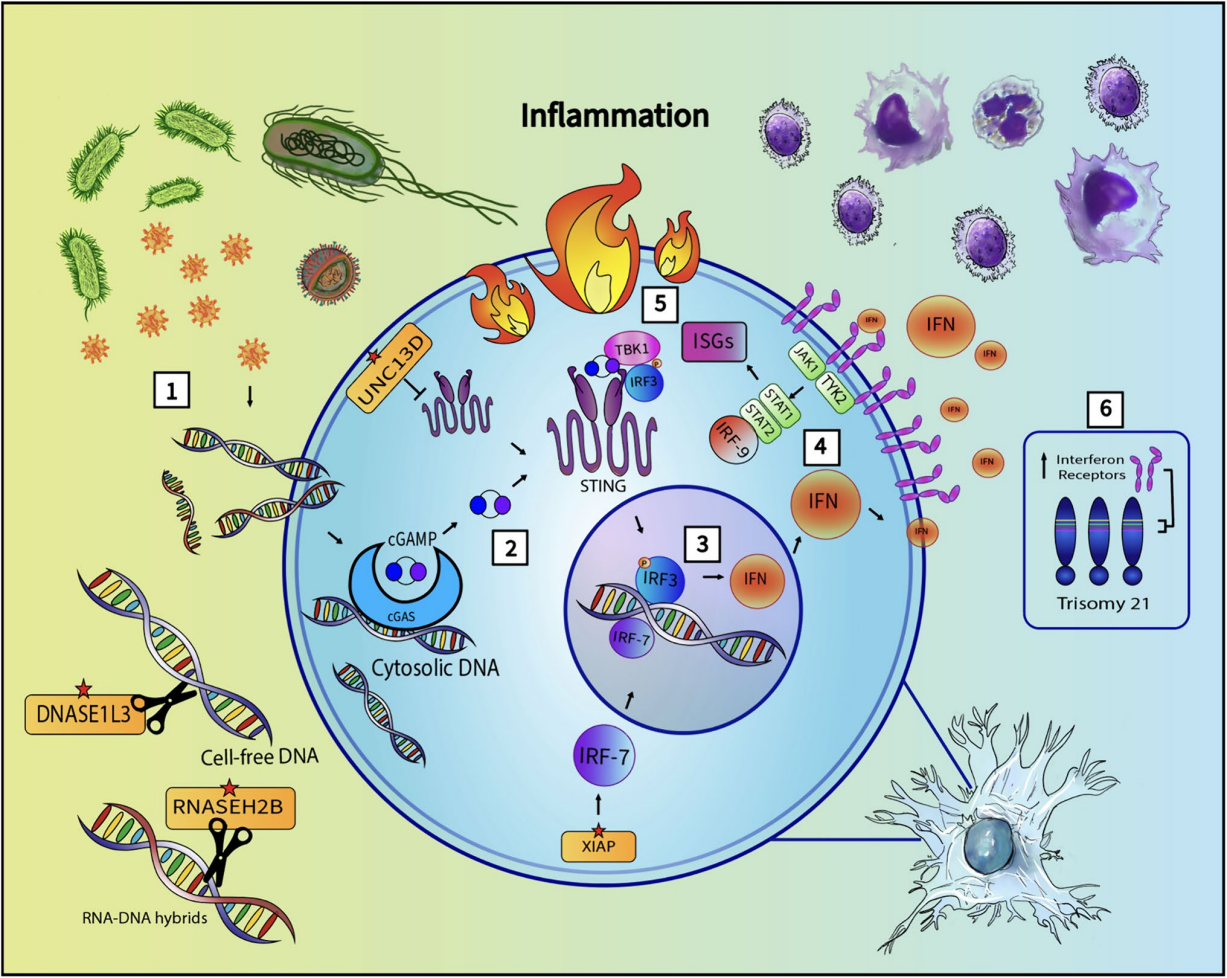
Interferon type-1 response. (1) Pathogens and damaged cells release nucleic acids. (2) The cytosolic DNA activates the cGAS-STING pathway, resulting in phosphorylation of interferon regulatory factor 3 (IRF3). (3) The activated IRF3 induces transcription of interferons in the nucleus. (4) Interferon type-1 binds to the heterodimeric IFNα/β receptor (IFNAR), activating the associated tyrosine kinases JAK1 and TYK2, which in-turn activate STAT1 and STAT2 transcription factors. (5) STAT homo/heterodimers, combined with IRF-9, activate the transcription of interferon-stimulated genes (ISGs), resulting in inflammation. (6) Chromosome 21 encodes four of the INFAR subunits. Trisomy of chromosome 21 results in increased expression of INFAR, resulting in an amplified JAK/STAT response to triggers. *Proteins encoded by genes harboring a pathogenic variant identified in DSRD are highlighted with a red star. UNC13D regulates the inflammatory response by inhibiting the oligomerization of STING. Deoxyribonuclease 1 like 3 (DNase1L3) degrades cell-free DNA, and Ribonuclease H2 (RNase H2) cleaves RNA-DNA hybrids, preventing cytosolic entry and accumulation of unprocessed nucleic acids

Trisomy 21 is known to cause an amplified interferon response [11]. The chromosome 21 carries genes encoding four out of the six interferon receptor subunits. The presence of an extra copy of chromosome 21 leads to increased gene dosage, thereby elevating the expression of interferon receptors [11]. The increased expression amplifies the JAK/STAT signaling pathway, culminating in the upregulation of interferon-stimulated gene (ISG) expression in response to various triggers individuals with DS (Fig. 1) [26]. Our study did not find any one specific type of trigger (e.g., infectious) being preferentially associated with the harboring of gene variants although it is possible that the low total number of individuals with triggers was not sufficient to detect any effect. Alternatively, this result could also be interpreted as the etiology of the biological stress to the system may be irrelevant and that any activation of the inflammatory response may be sufficient to cause an overactive signaling pathway to cause pathology. This will be an important area of future investigation.

It is important to note that all genes harboring pathogenic variants identified in our cohort encode proteins associated with interferon type-1 inflammatory response. The Unc-13 homolog D (UNC13D) protein regulates the inflammatory response by inhibiting the oligomerization of Stimulator of interferon genes (STING) [27]. Deoxy- ribonuclease 1 like 3 (DNase1L3) degrades cell-free DNA [28], and Ribonuclease H2 (RNase H2) cleaves RNA–DNA hybrids, preventing the inflammatory response triggered by cytosolic entry and accumulation of unprocessed nucleic acids [29]. X-linked inhibitor of apoptosis protein (XIAP) increases the stability of Interferon regulatory factor 7 (IRF7), a transcription factor that drives expression of type 1 interferons (Fig. 1) [30].

Furthermore, pathogenic variants in the identified genes are linked to interferonopathies, autoimmune, and autoinflammatory disorders such as Aicardi-Goutieres syndrome (AGS), familial hemophagocytic lymphohistiocytosis (HLH), systemic lupus erythematous (SLE), and inflammatory bowel disease (IBD). For instance, biallelic variants of *UNC13D* are associated with familial HLH type 3 [31–33]. Pathogenic variants in genes encoding RNase H2 subunits (*RNASEH2A, RNASEH2B, and RNASEH2C*) are known causes of AGS [34, 35]. In addition, heterozygous variants of *RNASEH2* are shown to be associated with SLE and increased risk of systemic autoimmunity [36]. Pathogenic variants of *DNASE1L3* are associated with familial SLE, marked by childhood-onset of anti-ds DNA positivity [37–39]. XIAP deficiency is marked by immune system dysfunction and a wide range autoinflammatory manifestations, including HLH and IBD [40]. Interestingly, serum analysis of individuals in our cohort with identified pathogenic variants was unremarkable and not consistent with any of the mentioned inflammatory conditions as noted in prior reports [6].

We speculate that deleterious variations in genes associated with interferon response may increase the likelihood of abnormal responses to common pathogens or stressors. Instances of rapid deterioration in response to common infections have been described in individuals with AGS and HLH, conditions arising from biallelic pathogenic variants in genes identified in this study [32, 34, 41]. This suggests that heterozygous variants in these genes might exacerbate the inflammatory response to various triggers, potentially contributing to the development of DSRD. This hypothesis is supported by the observation of a higher likelihood of preceding triggers and a more rapid decline to nadir of DSRD symptoms among individuals with variants.

**Fig. 2:**
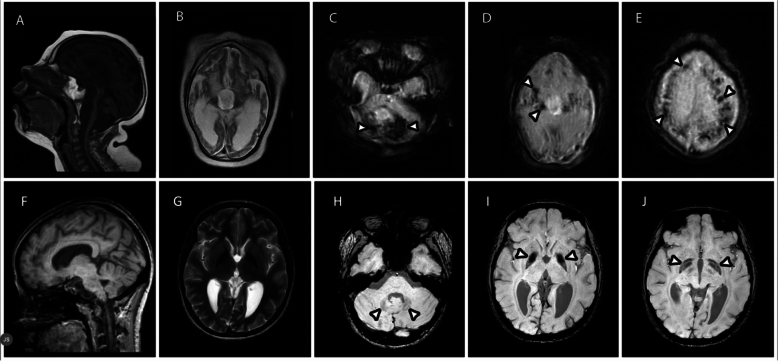
MRI images in a 4-year-old male with Aicardi-Goutieres syndrome (**A**–**E**) and an 18-year-old female with Down syndrome regression disorder (**E**–**I**). Sagittal T1-weighted (**A**, **E**), axial T2-weighted (**B**, **F**), and axial SWI (**C**–**E**, **G**–**I**) are shown. In AGS, there is diffuse cerebral and cerebellar atrophy with ex-vacuo ventriculomegaly (**A**, **B**), diffuse dystrophic calcification involving cerebellum (**C**), basal ganglia (**D**), and supratentorial cortex and white matter (**E**, arrowheads). In DSRD, there is mild cerebral atrophy (**F**, **G**). Dystrophic mineralization demonstrated in dentate nuclei of the cerebellum (**H**), basal ganglia (**I**, **J**, arrowheads)

Additional evidence comes from the neuroimaging abnormalities in DSRD, including susceptibility weighted imaging (SWI) findings of dystrophic mineralization of the basal ganglia, which resemble findings in AGS and HLH, albeit often more diffuse and extensive in the latter conditions (Fig. 2) [42, 43]. Individuals with variants were more likely to demonstrate abnormal neuroimaging findings compared to those without identified variants, although the total number compared was relatively small.

Interestingly, harboring variants did not correlate with an increased rate of immunotherapy responsiveness in this cohort. This may be secondary to the small sample size and the already notable rate of immunotherapy responsiveness in individuals with DSRD. However, it is noteworthy that all individuals with a pathogenic or likely pathogenic variant were immunotherapy responsive.

This study is not without limitation. Firstly, our analysis was retrospective in nature and is influenced by both selection and severity bias. Individuals evaluated or seeking second opinions at the host institution would be presumed to have more severe phenotypes. Nevertheless, the inclusion of individuals with severe phenotypes offers a unique opportunity to detect rare variants with potentially significant effect sizes [44]. Individuals who had exome sequencing were more likely to have private insurance, further introducing selection bias towards individuals with higher socioeconomic status. Compounded with selection and severity bias, generalizability of these findings is limited and should be interpreted with reservation. The rare nature of DSRD and financial limitations on obtaining exome sequencing were factors in lowering the power of this study as well.

The causality of identified variants requires validation through functional studies. The interface between phenotype and genotype is complicated and influenced by a variety of environmental, social and health factors, and while these genes are of interest, they may not be sufficient to cause DSRD. Further exploration of this subset of individuals may provide crucial insights into DSRD's pathophysiology. Further large-scale studies comparing whole genome sequencing and genome-wide methylation analysis in DSRD and DS controls are warranted to better understand the genetic and epigenetic changes associated with DSRD.

## Conclusion

A small subset of individuals with DSRD were observed to have pathogenic variants in genes of immune regulation. The significance of this finding is underscored by the established links between these genes and interferon-mediated disorders such as AGS and SLE. These results mark a promising area for future investigations into the intricate mechanisms underlying DSRD and may lead to new immune targeting treatments.

### Supplementary Information

Below is the link to the electronic supplementary material.

Supplementary file1 (DOCX 20 KB)

Supplementary file2 (DOCX 19 KB)

Supplementary file3 (DOCX 49 KB)

## Data Availability

Additional data regarding patients is available to qualified investigators pending IRB approval.
