# Supplementary material for: De novo variants in immune regulatory genes in Down syndrome regression disorder
Source: J Neurol. 2024 Jun 22;271(8):5567–76. doi: 10.1007/s00415-024-12521-y (PMC11319504; doi:10.1007/s00415-024-12521-y)
Supplement: Supplementary file 1 — Supplementary file1 (DOCX 20 KB) [file 415_2024_12521_MOESM1_ESM.docx]

| **Table S1. Clinical characteristic of individuals with pathogenic variants in comparison to those without variants** | | | | | |
| --- | --- | --- | --- | --- | --- |
|  | **Patient1 *(UNC13D)*** | ***Patient 2***  ***(XIAP)*** | **Patient 3**  ***(RNASEH2A)*** | **Patient 4**  ***(DNASE1L3)*** | **DSRD without variants**  **(n = 33)** |
| Sex | Male | Female | Male | Female | 51.5% Female |
| Race | White | Asian | White | White | 78.7% White  12.1% Black  6.1% Asian  3.6% other |
| Ethnicity | Hispanic | Not Hispanic | Not Hispanic | Not Hispanic | 18.2 % Hispanic |
| Age at Symptom Onset (years) | 11 | 13 | 15 | 20 | median (IQR):14.9 (12-16) |
| Age at Diagnosis (years) | 13 | 15 | 15 | 22 | median (IQR):16.2 (16-22) |
| Congenital Heart Disease (CHD) | No | No | Yes | No | 30.1% with CHD |
| Non-DSRD Autoimmune Disease | Hashimoto’s Thyroiditis | Celiac Disease  Type I Diabetes | Hashimoto’s hyroiditis  Juvenile Idiopathic Arthritis  Alopecia Areata  Vitiligo | Hashimoto’s hyroiditis  Celiac Disease | 42.4% with personal history of autoimmune disease |
| Prior Diagnosis of ASD | No | No | No | No | 18.1% with prior diagnosis of ASD |
| Trigger Present  *type* | Yes  Viral URI two weeks prior | Yes  Viral URI three weeks prior and recent death in family (grandfather) | Yes  Viral URI and pneumonia three weeks prior | No | 48.5% with preceding trigger |
| Weeks to Symptom Nadir | 2 | 2 | 4 | 3 | 3.5 (median) |
| Serum Cytokine Abnormalities | Yes | Yes | Yes | No | 39.3% with cytokine abnormalities |
| Other immune profiling abnormality | ANA +  TPO AB +  TG Ab +  Hypovitaminosis D  Leukopenia  Lymphopenia (ALC = 440) | ANA+  Leukopenia | TPO Ab +  TG Ab +  Hypovitaminosis D | TPO Ab +  Leukopenia  ESR Elevated | 12.1% ANA+  3% dsDNA+  42.4% TPO Ab +  30.1% TG Ab +  18.1% ESR Elevated  36.3% Hypovitaminosis D  12.1% Leukopenia  3% Lymphopenia |
| EEG Abnormal Abnormality | Yes (generalized slowing) | No | No | Yes (generalized slowing) | 24.2% |
| Neuroimaging Abnormality | Yes  (T2 signal prolongation throughout cortex, SWI signal abnormality in bilateral basal ganglia) | Yes  (Bilateral SWI signal abnormality in basal ganglia) | Yes  (SWI signal abnormality in the basal ganglia and dentate nuclei of cerebellum) | No | 30.3% with MRI abnormalities |
| CSF Abnormality | Yes  (Increased IgG Index of 0.84) | No | Yes  (Pleocytosis of 6 WBC, increased IgG Index of 0.84) | No | 18.1% with CSF abnormalities |
| Catatonia | Yes | Yes | Yes | Yes | 75.7% with catatonia |
| Immunotherapy Responsive (Specify) | Yes  (IVIg) | Yes  (IVIg) | Yes  (IVIg and Steroids) | Yes  (IVIg and Steroids) | 72.7% with immunotherapy responsiveness |
| BFCRS Score at Baseline | 40 | 36 | 43 | 18 | 33 (median) |
| BFCRS Score at 24 Weeks | 18 | 6 | 11 | 8 | 19 (median) |
| Decrease BFCRS | 22 | 30 | 32 | 10 | 14 (median) |
| NPI-Q Total Score at Baseline | 100 | 36 | 28 | 56 | 54 (median) |
| NPI-Q Total Score at 24 Weeks | 38 | 11 | 15 | 23 | 26 (median) |
| Decrease NPI-Q | 62 | 25 | 13 | 23 | 28 (median) |
| ALC: Absolute lymphocyte count, ANA: Antinuclear antibody, ASD: Autism Spectrum Disorder, BFCRS: Bush-Francis Catatonia Rating Scale, CSF: cerebrospinal fluid, dsDNA: double-stranded deoxynucleic acid, EEG: electroencephalogram, IVIg: intravenous immunoglobulins, MRI: magnetic resonance imaging, NPI-Q: Neuropsychiatric Inventory- Questionnaire, SWI: susceptibility weighted imaging, TG: Thyroglobulin, TPO: Thyroid peroxidase, URI: upper respiratory infection. | | | | | |
