# Supplementary material for: De novo variants in immune regulatory genes in Down syndrome regression disorder
Source: J Neurol. 2024 Jun 22;271(8):5567–76. doi: 10.1007/s00415-024-12521-y (PMC11319504; doi:10.1007/s00415-024-12521-y)
Supplement: Supplementary file 2 — Supplementary file2 (DOCX 19 KB) [file 415_2024_12521_MOESM2_ESM.docx]

| **Table S2. Clinical characteristics of patients with DSRD with variants of uncertain significance.** | | | | |
| --- | --- | --- | --- | --- |
|  | **Patient 5**  ***(BAZ1A)*** | **Patient 6**  ***(IRF7)*** | **Patient 7**  ***(SMARCAL1)*** | **Patient 8**  ***(LYST)*** |
| Sex  *Male*  *Female* | Male | Female | Male | Male |
| Race | White | Black | White | White |
| Ethnicity  *Hispanic or Latino*  *Not Hispanic or Latino* | Hispanic | Non-Hispanic | Hispanic | Non-Hispanic |
| Age at Symptom Onset (years) | 18 | 13 | 19 | 15 |
| Age at Diagnosis (years) | 18 | 15 | 22 | 16 |
| Congenital Heart Disease (CHD) | Yes | No | Yes | No |
| Non-DSRD Autoimmune Disease | Celiac Disease  Juvenile Idiopathic Arthritis | Celiac Disease | Hashimoto’s Thyroiditis  Celiac Disease  Type I Diabetes | Hashimoto’s Thyroiditis  Idiopathic Arthropathy |
| Prior Diagnosis of ASD | No | No | No | No |
| Trigger Present   - *If so, what type* | Yes  Graduation from high school (grade 12, USA) | Yes  Physical and Sexual Abuse three months prior | No | Yes  Moved homes and sibling moved away six months prior |
| Weeks to Symptom Nadir | 5 | 3 | 8 | 7 |
| Serum Cytokine Abnormalities | No | No | No | No |
| Other immune profiling abnormality (Specify) | ESR Elevated  Leukopenia  ANA+  dsDNA+ | ANA+  TPO Ab+  Hypovitaminosis D | TPO Ab+  TG Ab+  Leukopenia | ANA+  ESR Elevated  TPO Ab+  TG Ab+  Hypovitaminosis D |
| EEG Abnormal Abnormality | Yes  (generalized slowing) | No | Yes  (generalized slowing) | No |
| Neuroimaging Abnormality | No | Yes  (Bilateral SWI signal abnormality in the basal ganglia) | No | Yes  (T2 signal prolongation in the distal cortical white matter bilaterally) |
| Lumbar Puncture Abnormality | No | No | No | No |
| Catatonia | Yes | Yes | Yes | Yes |
| Immunotherapy Responsive (Specify) | Yes  (IVIg) | No | Yes  (IVIg and Steroids) | Yes  (IVIg) |
| BFCRS Score at Baseline | 24 | 24 | 30 | 12 |
| BFCRS Score at 24 Weeks | 10 | 22 | 16 | 7 |
| Decrease BFCRS | 14 | 2 | 14 | 5 |
| NPI-Q Total Score at Baseline | 44 | 72 | 38 | 30 |
| NPI-Q Total Score at 24 Weeks | 20 | 64 | 14 | 18 |
| Decrease NPI-Q | 24 | 8 | 24 | 12 |
| ALC: Absolute lymphocyte count, ANA: Antinuclear antibody, ASD: Autism Spectrum Disorder, BFCRS: Bush-Francis Catatonia Rating Scale, CSF: cerebrospinal fluid, dsDNA: double-stranded deoxynucleic acid, EEG: electroencephalogram, IVIg: intravenous immunoglobulins, MRI: magnetic resonance imaging, NPI-Q: Neuropsychiatric Inventory- Questionnaire, SWI: susceptibility weighted imaging, TG: Thyroglobulin, TPO: Thyroid peroxidase, URI: upper respiratory infection. | | | | |
