# Supplementary material for: De novo variants in immune regulatory genes in Down syndrome regression disorder
Source: J Neurol. 2024 Jun 22;271(8):5567–76. doi: 10.1007/s00415-024-12521-y (PMC11319504; doi:10.1007/s00415-024-12521-y)
Supplement: Supplementary file 3 — Supplementary file3 (DOCX 49 KB) [file 415_2024_12521_MOESM3_ESM.docx]

| **Table S3. Genes harboring variants identified in the study and associated conditions.** | |
| --- | --- |
| ***Gene*** | **Function** |
| ***BAZ1A*** (HGNC: 960) | Encodes a subunit of the ATP-dependent chromatin assembly factor (ACF). A variant of BAZ1A is previously reported in VACTERL associated with brain malformation, including agenesis of corpus callosum and absent septum pellucidum.^1^ |
| ***DNASE1L3*** (HGNC:2959) | Encodes Deoxyribonuclease 1L3, a member of the DNase family, involved in the fragmentation of plasma DNA. Short fragments of DNA exist in the circulation because of release of cellular DNA to the plasma upon cell death. A deficiency in DNASE1L3 can result in the buildup of unfragmented circulating DNA, triggering an autoimmune response, including the production of anti-dsDNA antibodies. Homozygous pathogenic variants of *DNASE1L3* are associated with familial SLE, marked by childhood onset of anti-ds DNA positivity and lupus nephritis.^2^ A heterozygous loss of function variant could also cause the disease phenotype.^3, 4^ Information on the remainder of genes could be found in Table S2. |
| ***IRF7*** (HGNC: 6122) | Encodes Interferon regulatory factor 7, a crucial regulator of expression of interferon type 1 genes in response to detection of viral components by PRR.^5^ It was initially identified in relation to EBV infection, as it is activated by the EBV latent membrane protein-1 (LMP1).^6^ Homozygous IRF7 variants have been associated with severe influenza infection and herpes simplex encephalitis.^7^ |
| ***LYST*** (HGNC: 1968) | Encodes lysosomal trafficking regulator LYST. Loss of function of LYST causes dysregulated phagosomal maturation. Biallelic pathogenic variants in *LYST* cause Chédiak-Higashi syndrome (CHS). Patients with CHS experience predilection for bleeding, partial albinism, immune deficiency with recurrent infections, risk of development of HLH, and progressive neurological manifestations such as early learning and cognitive deficits, progressive neurodegeneration, cerebral and cerebellar atrophy, dystonia, parkinsonism, and peripheral neuropathy.^8-10^ CHS presents as a spectrum of clinical phenotypes, and in compound heterozygous or monoallelic variant of *LYST*, milder and atypical symptoms could be observed.^9, 10^ |
| ***RNASEH2*** (HGNC: 18518) | Encodes Ribonuclease H2, an enzyme responsible for breaking down RNA within RNA/DNA hybrid molecules (R loops). RNase impairment results in the buildup of R-loops, causing DNA damage and triggering an immune response.^11^ Biallelic variants of RNase H2 subunits (*RNASEH2A, RNASEH2B*, and *RNASEH2C*) are the most common causes of Aicardi-Goutieres syndrome (AGS).^12^ In addition, heterozygous variants of *RNASEH2* are shown to be associated with Systemic Lupus Erythematous (SLE) and increased risk of systemic autoimmunity.^13^ |
| ***SMARCAL1*** (HGNC:11102) | SWI/SNF related, matrix associated, actin dependent regulator of chromatin, subfamily a like 1 (SMARCAL1): is a chromatin remodeling protein that has a crucial role in DNA repair. Biallelic variants in *SMARCAL1* are associated with Schimke immuno-osseous dysplasia, characterized by nephropathy/nephrotic syndrome, spondyloepiphyseal dysplasia resulting in short stature, and T cell immunodeficiency.^14, 15^ |
| ***UNC13D*** (HGNC: 23147) | Encodes MUNC13-4 protein, which is essential for cytolytic granule secretion and lymphocyte cytotoxicity.^16^ Biallelic variants of *UNC13D* are associated with familial hemophagocytic lymphohistiocytosis (HLH) type3, although monoallelic variants have also been described in association with the disease phenotype and NK granulation defect with decreased MUNC13-4 protein expression.^17-19^ |
| ***XIAP*** (HGNC: 592*)* | Encodes X-linked Inhibitor of Apoptosis, which is involved in the immune response through inhibition of caspases in natural killer T cells and mucosal-associated T cells. In addition, XIAP functions as a signal transducer for the Nod-like receptors NOD1and NOD2 as well as regulating the activation of the NLRP3 inflammasome.^20^ Nod-like receptors are members of pattern recognition receptors (PRR) which are crucial for the innate immune response through detection of pathogens within the cytoplasm.^21^ XIAP deficiency is marked by immune system dysfunction and a wide range autoinflammatory manifestations, such as HLH, inflammatory bowel disease (IBD), hypogammaglobulinemia, and increased susceptibility to infections.^20^ |

1. Weitensteiner V, Zhang R, Bungenberg J, et al. Exome sequencing in syndromic brain malformations identifies novel mutations in ACTB, and SLC9A6, and suggests BAZ1A as a new candidate gene. Birth Defects Res 2018;110:587-597.

2. Chan RWY, Serpas L, Ni M, et al. Plasma DNA Profile Associated with DNASE1L3 Gene Mutations: Clinical Observations, Relationships to Nuclease Substrate Preference, and In Vivo Correction. Am J Hum Genet 2020;107:882-894.

3. Yasutomo K, Horiuchi T, Kagami S, et al. Mutation of DNASE1 in people with systemic lupus erythematosus. Nat Genet 2001;28:313-314.

4. Al-Mayouf SM, Sunker A, Abdwani R, et al. Loss-of-function variant in DNASE1L3 causes a familial form of systemic lupus erythematosus. Nat Genet 2011;43:1186-1188.

5. Bourdon M, Manet C, Montagutelli X. Host genetic susceptibility to viral infections: the role of type I interferon induction. Genes Immun 2020;21:365-379.

6. Ning S, Pagano JS, Barber GN. IRF7: activation, regulation, modification and function. Genes Immun 2011;12:399-414.

7. Mogensen TH. IRF and STAT Transcription Factors - From Basic Biology to Roles in Infection, Protective Immunity, and Primary Immunodeficiencies. Front Immunol 2018;9:3047.

8. Westphal A, Cheng W, Yu J, et al. Lysosomal trafficking regulator Lyst links membrane trafficking to toll-like receptor-mediated inflammatory responses. J Exp Med 2017;214:227-244.

9. Sharma P, Nicoli ER, Serra-Vinardell J, et al. Chediak-Higashi syndrome: a review of the past, present, and future. Drug Discov Today Dis Models 2020;31:31-36.

10. Introne WJ, Westbroek W, Groden CA, et al. Neurologic involvement in patients with atypical Chediak-Higashi disease. Neurology 2017;88:e57-e65.

11. Cristini A, Tellier M, Constantinescu F, et al. RNase H2, mutated in Aicardi-Goutières syndrome, resolves co-transcriptional R-loops to prevent DNA breaks and inflammation. Nat Commun 2022;13:2961.

12. Mackenzie KJ, Carroll P, Lettice L, et al. Ribonuclease H2 mutations induce a cGAS/STING-dependent innate immune response. Embo j 2016;35:831-844.

13. Günther C, Kind B, Reijns MA, et al. Defective removal of ribonucleotides from DNA promotes systemic autoimmunity. J Clin Invest 2015;125:413-424.

14. Bansal R, Hussain S, Chanana UB, Bisht D, Goel I, Muthuswami R. SMARCAL1, the annealing helicase and the transcriptional co-regulator. IUBMB Life 2020;72:2080-2096.

15. Babaei AH, Inaloo S, Basiratnia M. Schimke Immuno-osseous Dysplasia: A Case Report. Indian J Nephrol 2019;29:291-294.

16. Feldmann J, Callebaut I, Raposo G, et al. Munc13-4 is essential for cytolytic granules fusion and is mutated in a form of familial hemophagocytic lymphohistiocytosis (FHL3). Cell 2003;115:461-473.

17. Rohr J, Beutel K, Maul-Pavicic A, et al. Atypical familial hemophagocytic lymphohistiocytosis due to mutations in UNC13D and STXBP2 overlaps with primary immunodeficiency diseases. Haematologica 2010;95:2080-2087.

18. Meeths M, Chiang SC, Wood SM, et al. Familial hemophagocytic lymphohistiocytosis type 3 (FHL3) caused by deep intronic mutation and inversion in UNC13D. Blood 2011;118:5783-5793.

19. Aricò M, Boggio E, Cetica V, et al. Variations of the UNC13D gene in patients with autoimmune lymphoproliferative syndrome. PLoS One 2013;8:e68045.

20. Mudde ACA, Booth C, Marsh RA. Evolution of Our Understanding of XIAP Deficiency. Front Pediatr 2021;9:660520.

21. Ratner AJ, Aguilar JL, Shchepetov M, Lysenko ES, Weiser JN. Nod1 mediates cytoplasmic sensing of combinations of extracellular bacteria. Cell Microbiol 2007;9:1343-1351.
